# Effect of missing data on multitask prediction methods

**DOI:** 10.1186/s13321-018-0281-z

**Published:** 2018-05-22

**Authors:** Antonio de la Vega de León, Beining Chen, Valerie J. Gillet

**Affiliations:** 1 Information School, University of Sheffield, Regent Court, 211 Portobello, Sheffield, S1 4DP UK; 2 Department of Chemistry, University of Sheffield, Dainton Building, Brook Hill, Sheffield, S3 7HF UK

**Keywords:** Multitask prediction, Sparse data sets, Missing data, Deep neural networks, Macau

## Abstract

There has been a growing interest in multitask prediction in chemoinformatics, helped by the increasing use of deep neural networks in this field. This technique is applied to multitarget data sets, where compounds have been tested against different targets, with the aim of developing models to predict a profile of biological activities for a given compound. However, multitarget data sets tend to be sparse; i.e., not all compound-target combinations have experimental values. There has been little research on the effect of missing data on the performance of multitask methods. We have used two complete data sets to simulate sparseness by removing data from the training set. Different models to remove the data were compared. These sparse sets were used to train two different multitask methods, deep neural networks and Macau, which is a Bayesian probabilistic matrix factorization technique. Results from both methods were remarkably similar and showed that the performance decrease because of missing data is at first small before accelerating after large amounts of data are removed. This work provides a first approximation to assess how much data is required to produce good performance in multitask prediction exercises.

**Electronic supplementary material:**

The online version of this article (10.1186/s13321-018-0281-z) contains supplementary material, which is available to authorized users.

## Introduction

Drug discovery has been changing focus for the last few years. The target-based approach, which has dominated the field for many years, is currently giving way to a more systems-based focus, boosted by heavy investment and research in omics science. In this framework, individual targets are replaced by molecular pathways with phenotypic, or cell-based, responses [1] as the optimization targets. Phenotypic screening offers several advantages over the one-target approach such as providing a biological response that is physiologically relevant. It has great potential to identify first-in-class drugs, however, determining the mechanism of action following a phenotypic screen is challenging. At the same time polypharmacology, which refers to the binding of chemical compounds to more than one target, has also been intensively studied [2]. The aim of these approaches is to identify multiple biological effects simultaneously, to better assess the selectivity profile of a compound across a range of related targets as well as potential side effects through off-target binding. One target family where these new approaches have informed recent drug discovery efforts is kinases. Kinases typically have very similar binding pockets [3] and many compounds originally thought to be selective kinase inhibitors later turned out to inhibit several kinases [4]. Today, many large kinase profiling exercises have been conducted to better assess the activity profile of kinase inhibitors [5].

In phenotypic screening and polypharmacology studies, the focus is on the biological response of compounds to a set of targets. Multitask machine learning methods are suitable in these scenarios, because they are able to predict several outputs with a single model. Data sets used for multitask prediction studies ought to be (near) complete, that is, each compound has been tested across the full set of targets. However, it is normally not possible to test so broadly in a cost-effective manner; leading to sparse data sets where not all molecules have been tested on all assays. This problem is exacerbated in academia, where data sets are usually assembled using public data sources such as PubChem [6] or ChEMBL [7]. The effect of missing activity information on the performance of multitask prediction has not been intensively studied in the chemoinformatics field. In many applications, missing activity records are assumed to be inactive, which may lead to false negatives [8]. Additionally, published guidance on how to best curate data for predictive modelling provides little information on how to handle missing activity data [9, 10].

Multitarget data sets can be used as the basis to predict novel molecules with polypharmacological properties. Currently, deep neural networks (DNNs) are gaining fame in drug discovery because of their multitask capabilities. These models should be able to discover molecules with specific activity profiles. DNNs have previously been used to perform large-scale predictive efforts on ChEMBL activity sets [11] and PubChem assays [12, 13]. They have also outperformed more traditional approaches such as Random Forest [14] and Naïve Bayes in recent competitions like the Tox21 challenge [15] and the Kaggle competition organized by Merck [16]. Current research focus on the applicability of these multitask capabilities for pharmaceutical companies [17]; as well as understanding the strengths and limitations of information sharing between tasks in multitask prediction [18]. However, DNNs are not the only multitask machine learning technique available. Ensemble tree methods, similar to Random Forest, have been modified to improve their performance in multitask scenarios [19, 20] and techniques based on Bayesian probabilistic matrix factorization have been applied to multitask chemoinformatics problems [21].

It is generally stated that deep learning methods like DNNs require large amounts of data [22]. The corollary from that statement is that more data produce better results. However, there has been little research into how sensitive these methods are to sparse data sets such as those currently being assembled in drug discovery efforts. Previous analyses have mainly focused on the effect of noisy data, especially in the context of high throughput screening data [23–25]. In these analyses, labels of some compounds were switched from active to inactive and vice versa.

In order to explore the effect of missing data on multitask prediction techniques, we assembled complete multitarget data sets to perform activity prediction based on both regression and classification. These data sets were made progressively sparser by removing activity records and the models re-learnt. Predictive performance of the models derived from the sparse data sets were compared with models learnt from the complete data sets to assess how much performance was lost through data removal. Three data removal models were compared, where individual activity labels, whole compounds, or whole assays were removed. Estimates were then determined for the point at which further data collection would not bring large improvements in performance. We compared DNNs to Macau, an alternative multitask prediction method, to test if the robustness of DNNs to data sparsity in this scenario is due to their multitask or their deep learning nature. Additionally, we also compared these methods to Random Forest, but implementation details restricted the analyses that could be performed on this technique.

## Materials and methods

### Datasets

In order to test prediction performance with respect to increasing data sparsity, we needed complete data sets where compounds had been tested consistently across a set of assays. We investigated two data sets: the PKIS data set, which was used for regression; and a data set extracted from PubChem, which was configured as a classification. For both data sets, SMILES strings [26] were obtained from the respective repositories. The molecules were standardized using MOE’s [27] wash function accessed through KNIME [28]. After standardization, Morgan fingerprints of radius 2 (equivalent to ECFP4 [29]) hashed to 1024 bits were computed using RDKit [30] in Python [31]. These fingerprints were used to represent molecules in the machine learning methods. Molecules that could not be read by MOE or RDKit were removed. The data sets are made available on an online repository (see Declarations section for details).

The PKIS data set [32] was provided by GSK to ChEMBL to promote the development of selective kinase probe compounds [33]. It consists of percent inhibition values for 367 compounds in 454 kinase assays. The majority of these assays were performed at Nanosyn, and a small fraction were performed by Frye’s Lab at University of North Carolina at Chapel Hill. In cases where several inhibition percent values were provided for individual compound-assay combinations, the mean of all reported values was used as the final value. Additionally, in 87 compound-assay combinations no activity value was provided, representing 0.05% of the activity profile matrix. These values were left empty.

The second data set was assembled using PubChem assays. We followed a previous report, where a set of 243 assays was selected to generate a public high-throughput screening fingerprint (HTSFP) [34]. The data were combined using the CIDs provided by the assays. The activity outcome was used as the activity label. Only ‘active’ and ‘inactive’ records were considered, and ‘inconclusive’ values were ignored. If a compound had more than one annotation for the same assay, and the annotations were different, that compound was also ignored. We used this large data set to generate two smaller subsets. For each subset, we chose a number of assays (five and ten) with the largest number of active molecules. We combined all records across the selected assays to generate a compound-assay matrix. Compounds were excluded if they were not active at least in one assay. The subset with five assays (HTSFP5) had 49,713 compounds while the set with 10 assays (HTSFP10) had 56,892 compounds. Table 1 describes the assays that were selected for HTSFP5 (the first five) and HTSFP10 (all assays in the table).

**Table 1:** Information for selected assays from PubChem

AID	Actives	Title	Assay type
2314	36968	Cycloheximide Counterscreen for Inhibitors of Shiga Toxin	Cell-based
1814	21686	MLPCN Alpha-Synuclein 5′UTR—5′-UTR binding—activators	Cell-based
743279	17142	Inhibitors of Inflammasome Signaling: IL-1-β AlphaLISA Primary Screen	Cell-based
504652	11249	Antagonist of Human D 1 Dopamine Receptor: qHTS	Cell-based
485346	10019	uHTS for Inhibitors of Mdm2/MdmX interaction	Cell-based
652054	9080	qHTS of D3 Dopamine Receptor Antagonist: qHTS	Cell-based
588726	8214	Inhibitors of the fructose-bisphosphate aldolase (FBA) of M. tuberculosis	Biochemical
2796	7988	Activators of the Aryl Hydrocarbon Receptor (AHR)	Cell-based
463190	7317	uHTS for inhibitors of tim10-1 yeast	Cell-based
687014	6834	Agonists of the DAF-12 from the parasite H. glycines (hgDAF-12)	Cell-based

For each selected assay the assay ID (AID), the number of active molecules, the title of the assay and the assay type are reported

### Simulating sparse data sets

Once complete data sets were assembled, they were used as a basis to simulate sparse data sets. The data sets were split randomly into training and test sets with a 3:1 ratio. For the training set, increasing numbers of activity labels (from no labels to all labels) were removed using three different removal models. In the first model (*label removal*, Additional file 1: Figure S1A), individual activity labels were randomly chosen and removed. This process maintained the size of the activity matrix but made it sparse (it generated empty cells in the matrix). For the second model (*compound removal*, Additional file 1: Figure S1B), whole compounds were removed at random. The third model (*assay removal*, Additional file 1: Figure S1C) removed whole assays at random and was only applied to the PKIS data set, as the number of assays in the HTSFP subsets was deemed too small. In the second and third models, the size of the activity matrix became smaller, as compounds or assays with no information were discarded, but the matrix was still complete. For the test set, no activity label removal was performed.

### Multitask prediction

Three machine learning methods were used to predict activity labels. All methods are able to produce multitask predictions, in which all assays are predicted with one model. Therefore, the model generates a profile of predicted values.

Deep neural networks are machine learning methods based on large numbers of simple, non-linear units called neurons [22]. We used fully connected DNNs, where neurons are organized in layers and all neurons in one layer are connected to all neurons in the next layer. These neurons accept a set of input values, perform a weighted sum and then use a non-linear activation function whose output is passed on to the next layer. We used the rectified linear unit (ReLU) as the activation function. Training a neural network is done through backward propagation with a gradient descent algorithm. Given a cost function that is minimized during training, the gradient around the current parameter values is estimated and new values of parameters are chosen that reduce the cost function. These gradients are first computed for the output layer and then are propagated backwards. We used the Adagrad optimizer function with a learning rate value of 0.05 (the default settings) to train all the networks. DNNs were implemented using the Python library Tensorflow [35].

Macau is a machine learning technique based on Bayesian probabilistic matrix factorization (BPMF) [21]. BPMF is a method frequently used in recommender systems, where the preference of a user for a specific item is predicted. It gained fame when matrix factorization methods were used in the winning submission to the Netflix prize [36]. In this competition, Netflix made available more than 100 million ratings that around 480,000 users gave to more than 17,000 movies, leading to a data set that was very sparse, containing ratings for only 1.2% of all user-movie combinations. Macau is a regression technique specifically designed to deal with sparse data sets. Because this is one of the first applications of this technique in chemoinformatics, we provide an abridged explanation of the technique below based on the details provided in ref. [21].

Matrix factorization is the process where a matrix is decomposed into two matrices linked through a latent space of predefined dimension:

$$X \approx UV^{T}$$

where $X$, $U$, and $V$ are matrices of dimensions $n \times m$, $n \times k$, and $m \times k$, respectively. $n$, $m$, and $k$ are the number of rows, columns, and latent dimensions, respectively. This process is well understood for complete matrices, and is the basis of singular value decomposition and principal component analysis.

Probabilistic matrix factorization expands the scope of this technique to incomplete matrices, allowing it to predict empty values in the matrix. It turns the matrix decomposition into an optimization problem formulated as:

$$\mathop {\hbox{min} }\limits_{{\varvec{u},\varvec{v}}} \mathop \sum \limits_{{\left( {i,j} \right) \in I_{X} }} \left( {X_{ij} - \varvec{u}_{i}^{ } \varvec{v}_{j}^{T} } \right)^{2}\,+ \,\lambda_{u}\!\parallel\!\varvec{u}\!\parallel_{F}^{2}
\,+ \,\lambda_{v} \!\parallel\!\varvec{v}\!\parallel_{F}^{2}$$

where $X_{ij}$ is the observed value, $\varvec{u}_{i}$ and $\varvec{v}_{j}$ are the latent vectors of the $i$th row and $j$th column, $I_{X}$ is the set of matrix cells with filled values, $_{F}$ is the Frobenius norm, and $\lambda_{u}$ as well as $\lambda_{v}$ are regularization parameters.

BPMF, in turn, improves on the optimization by modelling the latent matrices as priors using Gaussian distributions. These priors are based on a set of means ($\mu_{u}$ and $\mu_{v}$) and precision matrices ($\varLambda_{u}$ and $\varLambda_{v}$), as well as Normal and Normal-Wishart hyperpriors. BPMF uses Markov chain Monte Carlo sampling, specifically Gibbs sampling, to perform its inference over parameters and latent vectors. Additionally, BPMF provides a distribution of values, rather than a single value, during the prediction.

Macau adds to BPMF methods by integrating side information, among other improvements. Side information are features related to the entities represented by the rows or columns. In ref. [21], the authors used substructure fingerprints as side information for compounds and protein sequence features as side information for the targets. This information is combined into the mean of the Gaussian priors to be used during the model training.

Macau was implemented using the Python package Macau. To make results as comparable to DNN as possible, only molecules were given side information in the form of fingerprints. Assays were not provided with side information. The final predicted value was the mean of the distribution of values predicted. To perform classification on the HTSFP sets, ‘active’ and ‘inactive’ labels were transformed to integers and predicted values were rounded to the nearest integer and assigned the corresponding label.

Random Forest is a tree ensemble method. Several decision trees are constructed using a subset of the compounds and the fingerprint bit positions. The final output combines the individual predictions of each tree. Random Forest was implemented using the Python package scikit-learn. This implementation could not train the model if there was missing training data. For the application of Random Forest to the PKIS data set, which contained 87 missing activity labels in the original data, missing values were imputed using the average activity of the assay. Because the Random Forest could not be applied to the data sets generated using the label removal model, it was only used with the compound removal model.

### Performance measurements

For each machine learning method applied, several performance measures were calculated. In all cases, performance measures were calculated per assay. For regression models, the square of the correlation coefficient ($\rho^{2}$), the coefficient of determination ($R^{2}$), the mean absolute error ($MAE$), and the root mean square deviation ($RMSD$) were calculated. The formulae can be found in the Supplemental Information.

For classification models, the precision, the recall, the F_1_ score, and the Matthews correlation coefficient ($MCC$) were calculated. The formulae can be found in the Supplemental Information.

## Results

### Characterization of the data sets

The PKIS data set is a kinase profiling data set containing 367 compounds. We computed Tanimoto similarity values between all compound pairs. Similarity values varied between 0.02 and 1, with an average of 0.15 and a median of 0.13. The number of bits present in the fingerprints varied between 26 and 88, with a mean value of 52.5 and median value of 52. The percent inhibition values for all 454 kinase assays ranged from − 77 to 130, and 80% of the values were between 0 and 100.

The HTSFP data set was assembled from PubChem assays, following a previous publication. In this analysis, we chose the ten assays with largest number of actives. In the case of HTSFP5, the ratio of actives to inactives per assay varied from 2.9 to 0.25, while for HTSFP10 the ratios were generally lower, from 1.9 to 0.14. For both subsets, the average Tanimoto similarity between all pairs of molecules was 0.14 and the median was 0.13. The minimum, maximum, and median numbers of bits present was also the same in the two subsets; 12, 43, 102, respectively.

### Testing the effect of missing training data in multitask prediction

First, an exploratory analysis was performed on DNN, Macau, and Random Forest to assess the effect of hyperparameter selection on performance (data not shown). For DNN, the hyperparameters that were varied included number of hidden layers, number of neurons per layer, amount of dropout in hidden layers, number of training steps, activation function of neurons in the hidden layer, and size of the mini-batch during training. For Macau, the main hyperparameters studied were the number of samples in the training, the number of samples to burn in, and the size of the latent space. For Random Forest, the number of trees, the maximum number of features, and whether bootstrap was used during tree generation were varied. The specific values tested for each method and data set can be found in the Supplemental Information (Additional file 1: Tables S1–S6). In our tests, the ReLU activation function outperformed the sigmoid function consistently on DNNs. Therefore, it was used for all DNNs. This result was consistent with previous analysis [16].

Based on the results of the exploratory analysis, 10 hyperparameter sets were chosen for each method and data set. All hyperparameter sets can be found in the Supplemental Information (Additional file 1: Tables S7–S12). For each hyperparameter set, several predictive models (100 for PKIS and 39 for HTSFP subsets) were built using increasingly sparse training data (label removal model, Additional file 1: Figure S1A) as well as one model using the complete training data. The full results of all generated models are provided in an online repository (see Declarations for details). It is important to emphasize that we were not interested in achieving the highest possible performance for a model. Rather, we were interested in how the performance progresses as increasing amounts of training data were removed.

**Fig. 1:**
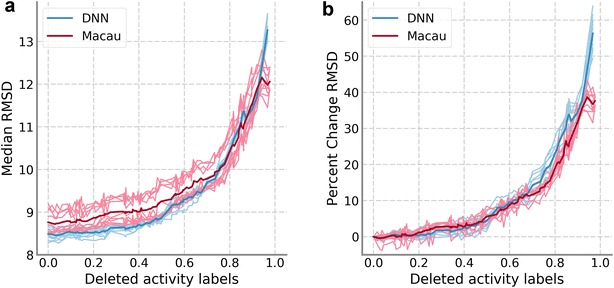
Results of training data sparseness on PKIS data set. **a** Median RMSD values for DNN (blue) and Macau (red). The light colored traces correspond to the ten sets of hyperparameters, while the dark colored trace is the average of the ten light colored ones. **b** The RMSD values are scaled relative to the performance of the model on the complete training set; the color scheme is the same as (**a**)

The results on the PKIS data set can be found in Fig. 1. We focused the regression analysis on the RMSD results; however, the trends were very similar for the other measures calculated (results for other performance measures are available in the online repository). Figure 1 displays the median of the RMSD values of all assays, DNN values in blue and Macau values in red, compared to the proportion of training data removed. In Fig. 1a, the median RMSD values are shown while in Fig. 1b values are scaled relative to the performance for the complete training set. The results for the ten hyperparameter sets are shown in a lighter color while the average over the ten sets is shown with a darker color. Overall, results for Macau were slightly worse than for DNNs when a small amount of activity labels were removed. However, the performance progression in relative terms was very similar for both machine learning techniques applied, as well as between all hyperparameters sets. On average, the median RMSD increase slowly at first; reaching a 10% increase only after 60% of the training set is removed. However, the increase in RMSD accelerates steeply afterwards. For models where more than 98% of the data were removed, the methods were not able to provide predictions because one or more assays had no activity annotation left. This is the reason why the trend lines do not extend to the full range (0.0–1.0) of data removal values tested.

**Fig. 2:**
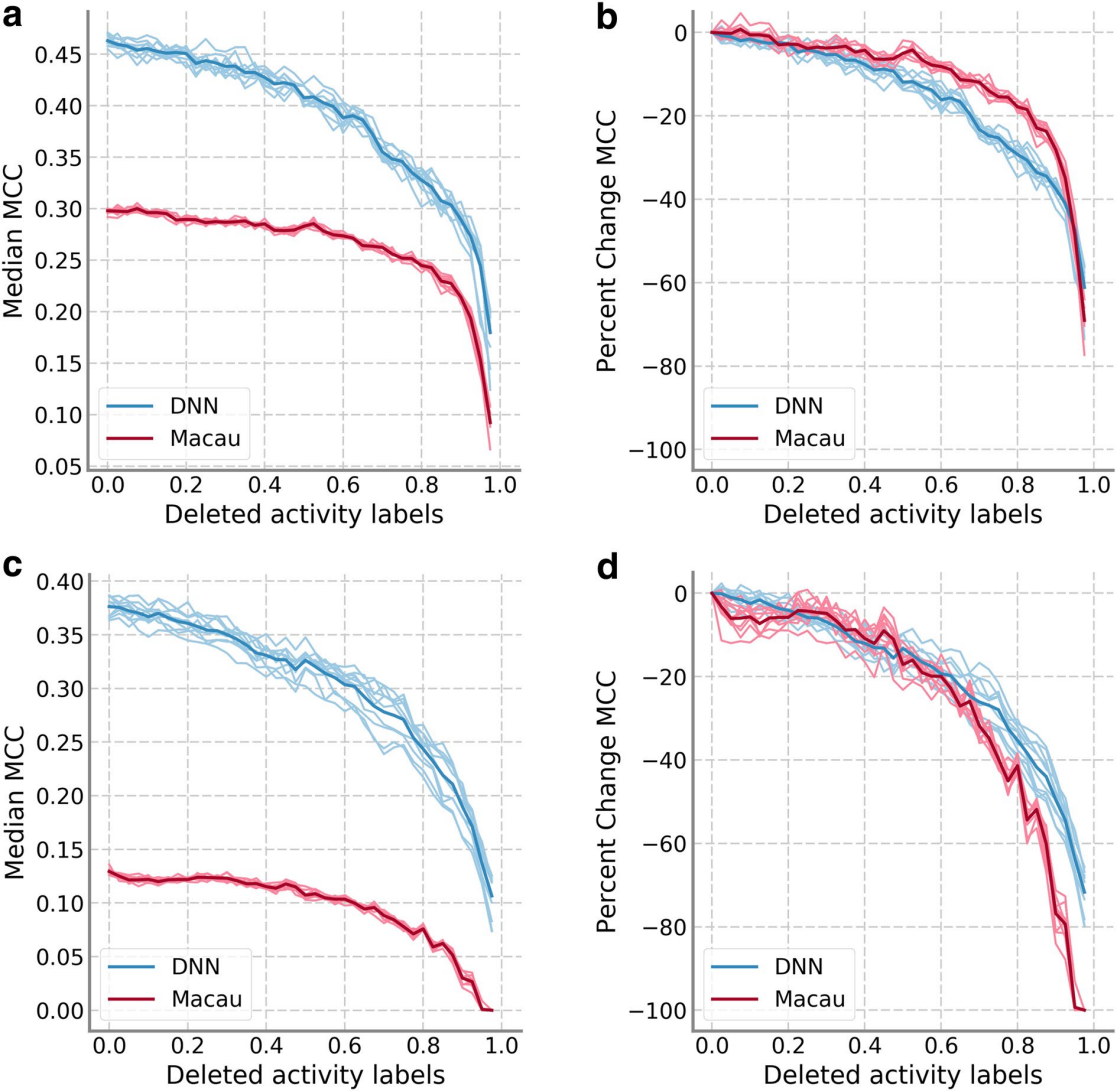
Results of training data sparseness on HTSFP subsets. **a** Median MCC values for DNN and Macau on the HTSFP5 subset. **b** The median MCC values for HTSFP5 are scaled relative to the performance of the model on the full training set. **c** Median MCC values on the HTSFP10 subset. **d** The median MCC values for HTSFP10 are scaled relative to the model with full training set. The color scheme follows the description in Fig. 1

Figure 2 shows results for the two subsets of the HTSFP data set. For classification problems, we focused the analysis on the MCC results but the trends for other performance measures were very similar. Figure 2 displays the median of the MCC values of all assays, following the color scheme of Fig. 1. Figure 2a, b focus on HTSFP5 while Fig. 2c, d focus on HTSFP10. In this analysis, performance of Macau was noticeably worse than DNN. This is likely because Macau is a regression technique that we have adapted to the task of classification through the use of threshold values as described in the Methods. However, performance progression was still similar to that seen in Fig. 1. Performance values decrease slowly for low amounts of data removed before decreasing sharply when most (≈ 80%) of the data was removed. Looking at absolute values (Fig. 2a, c) Macau’s progression might seem slower, but that could be attributed to its lower starting MCC value. When percentage changes were compared (Fig. 2b, d), the difference in progression between DNN and Macau was less severe.

### Controlling for randomness in training data

A control calculation was performed to test that the performance progression observed was not due to the specific data partitions performed. Four different seed values were generated and used to perform (a) the training/test split and (b) the removal of random activity labels. This generated 16 different runs where the training data fed to each model were different. For each run, 101 predictive models were built at different degrees of sparseness using the first set of hyperparameters for each method on the PKIS data set. Results are shown in Additional file 1: Figure S2, where DNN is shown left and Macau is shown right. In each plot, the relative median RMSD of all 16 different runs are shown, that is, the values are relative to the performance seen for the complete training set. The training/test split seed is represented using color, while the label removal seed is represented using line style. Some difference in absolute performance can be seen for the different training/test splits, however, the performance progression across all models follows a similar trend with a gradual increase in RMSD up to 60% of the data being removed.

The same analysis was performed on data sets HTSFP5 and HTSFP10, with 40 models trained for the same 16 combinations of seed values using the first set of hyperparameter values. Results are shown in Additional file 1: Figure S3, which uses the same representation of seed values as Additional file 1: Figure S2. Similar to the PKIS data set, each seed combination led to a very similar performance progression. Macau results on the HTSFP10 set show the largest variations. As discussed previously, this could be attributed to its lower absolute MCC values, such that small variations in median MCC resulted in larger percentage change values. These results show that the observed effects are independent of the specific data used for training.

### Comparison of different data removal models

Further control calculations were performed by comparing the three data removal models: label removal, compound removal, and assay removal. In all cases, the number of activity labels was reduced. However, in the case of the compound and assay removal models, the data matrix became smaller, as compounds or assays with no activity annotation were discarded, but was complete. On the other hand, the label removal model led to data matrices that were sparser but kept the original size. The assay removal model was only applied to the PKIS data set because the number of assays on the HTSFP subsets was considered too small.

**Fig. 3:**
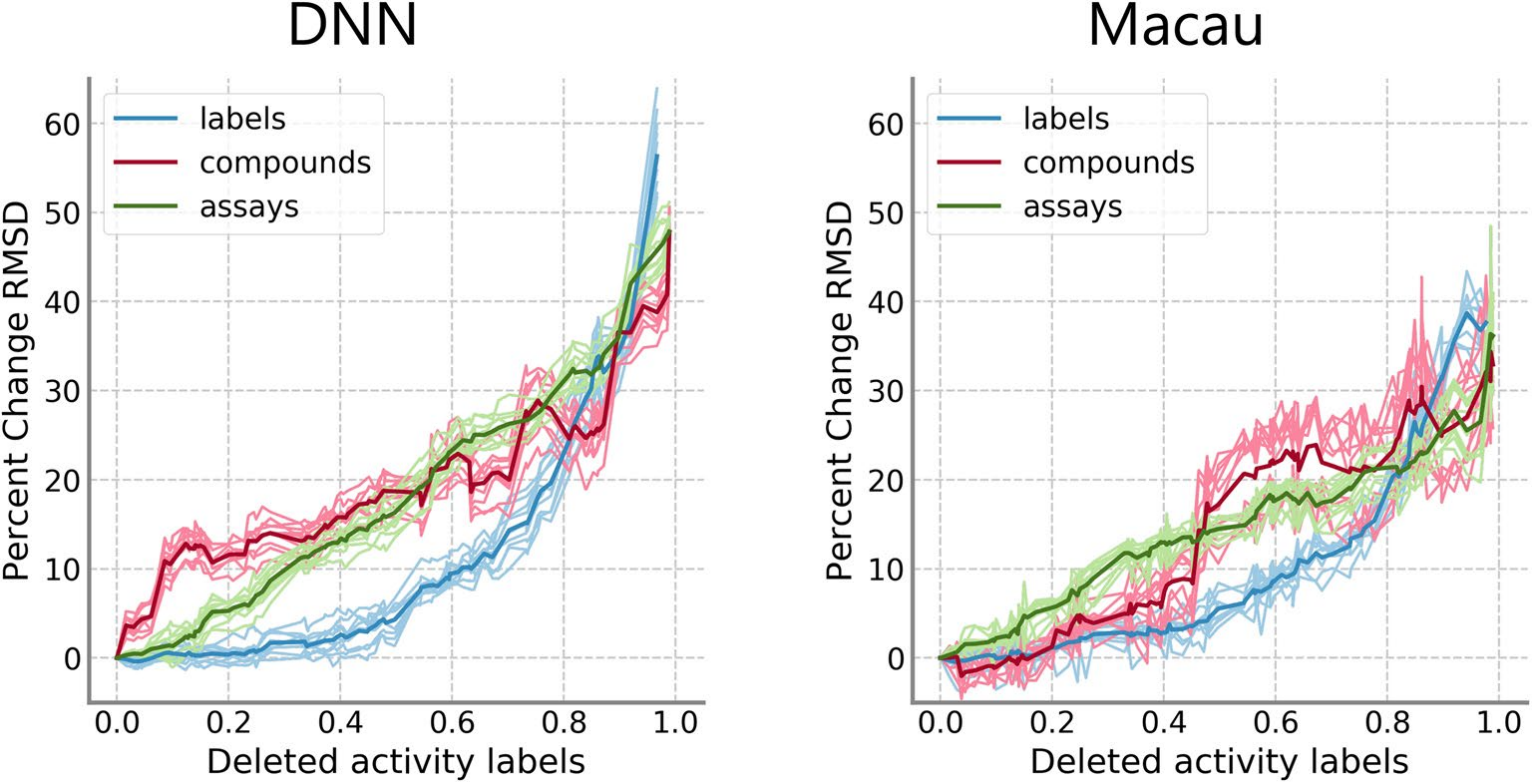
Comparison of removal models on PKIS data set. Median RMSD values relative to the model with complete training data are shown. Results from models that removed individual activity labels are shown in blue, results from models that removed whole compounds are shown in red, and results from models that removed whole assays are shown in green. Results for DNN (left) and Macau (right) are shown independently

**Fig. 4:**
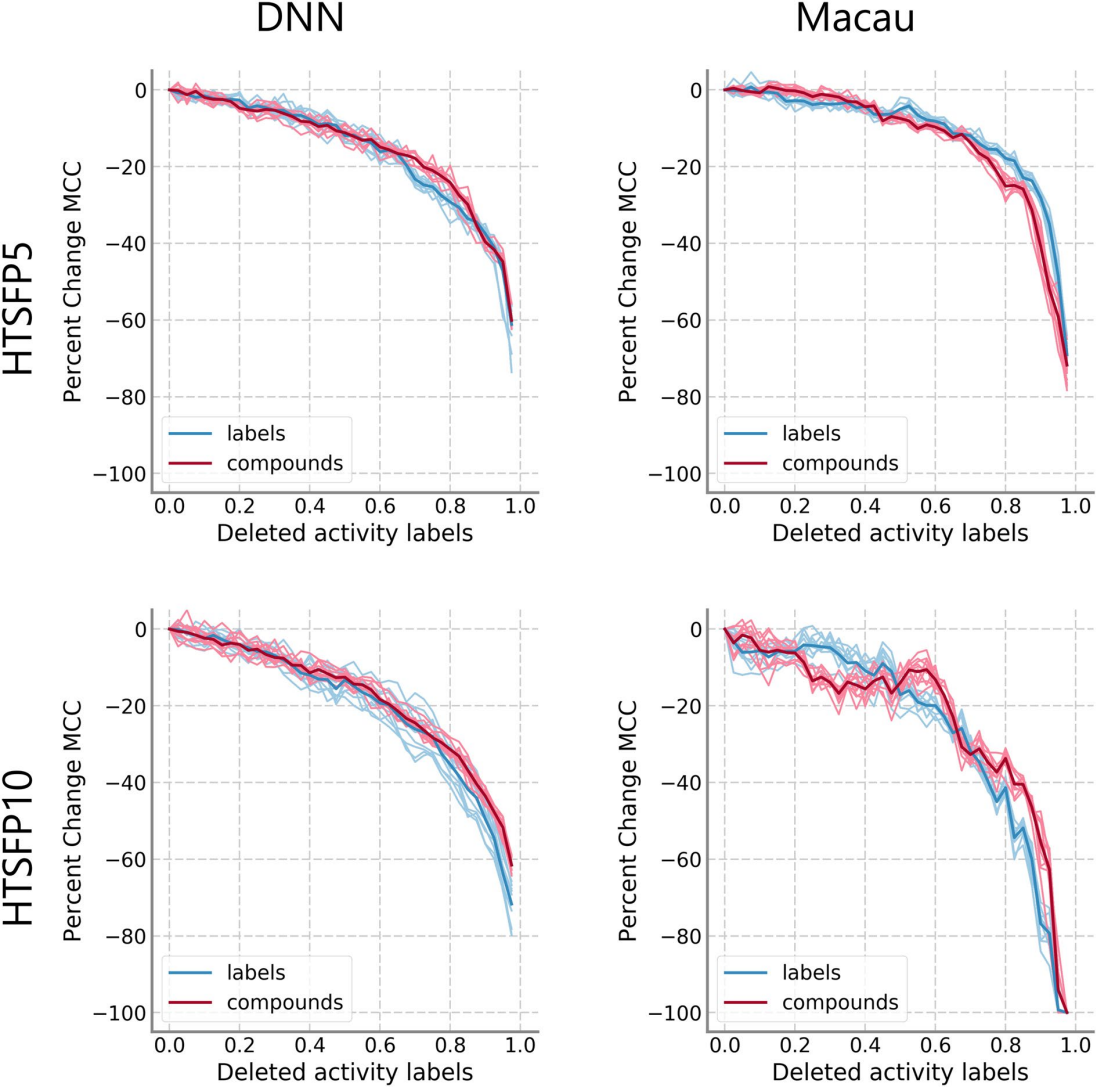
Comparison of removal models on HTSFP subsets. Median MCC values relative to the model with complete training data are shown. Results from models that removed individual activity labels are shown in blue, while results from model that removed whole compounds are shown in red. Results for DNN (left) and Macau (right), as well as results for HTSFP5 (top) and HTSFP10 (bottom), are shown independently

Similar to the first analysis, 101 models were trained for all 10 hyperparameter sets for each method and data removal model on the PKIS. Results are shown in Fig. 3 individually for DNN and Macau. Relative median RMSD values for the label removal model are shown in blue, while red is used for the compound removal model and green is used for the assay removal model. Differences between the compound and label removal models were more pronounced for DNNs, where there was a sharp difference in the progression from the very beginning. For Macau, differences became accentuated after 40% of the training data was removed. For the assay removal model, there is a linear decrease in performance, contrasting greatly to the performance progression of the label removal model. Removal of either whole compounds or whole assays generally led to worse performance. However, this trend was not observed on the HTSFP5 and HTSFP10 data sets (Fig. 4). For these data sets, performance progression between the label removal model and the compound removal model was very similar. There were not large differences in the results between DNN and Macau. The assay removal model was not applied because of the low number of assays in this data set, as previously mentioned.

**Fig. 5:**
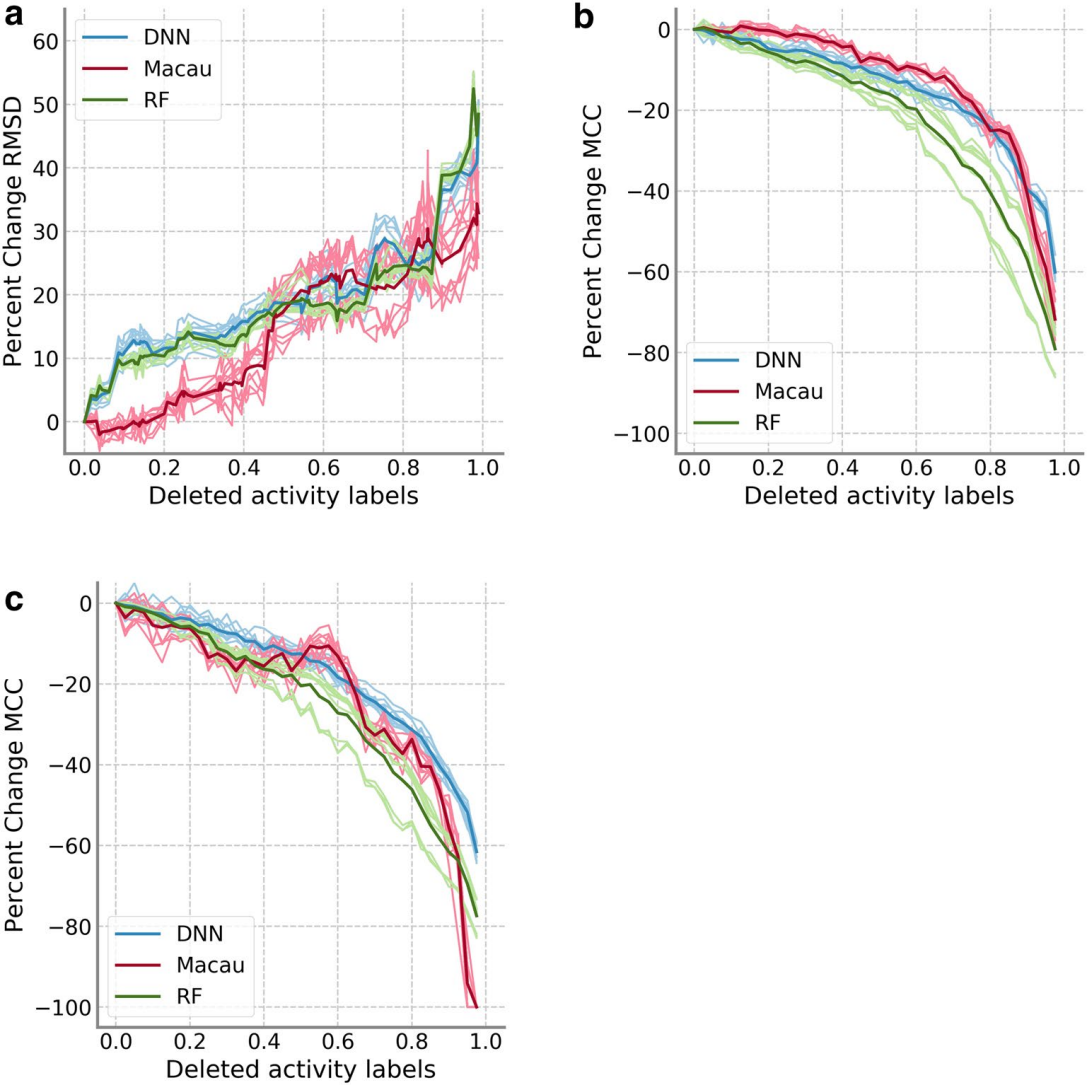
Comparison of Random Forest to DNN and Macau. **a** Median RMSD values relative to the model with complete training data for the PKIS data set are shown. **b**, **c** Median MCC values relative to the model with complete training data are shown for HTSFP5 (**b**) and HTSFP10 (**c**). Results for DNN are shown in blue, red for Macau and green for Random Forest

The compound removal model allowed the comparison of DNN and Macau to Random Forest for all data sets. Figure 5 shows the performance of DNN (blue), Macau (red), and Random Forest (green) for the PKIS and HTSFP data sets when whole compounds are removed. The performance progression of Random Forest was very similar to DNN on the PKIS data set (Fig. 5a). For the HTSFP data set, the decrease in performance was faster than either Macau or DNN (Fig. 5b, c). However, there was still an acceleration of the performance degradation as the amount of training data removed increased. Random Forest also showed larger variability between the results of the different hyperparameter sets than the other techniques. This variability is related to the large effect that some hyperparameters, such as whether bootstrap is used to construct the trees or the maximum number of features to use, have on the model performance.

## Discussion

We have analysed the effect of missing data on the performance of different multitask prediction methods. Our results showed that the performance decreases gradually as progressively larger amounts of data were removed from the training set. Indeed it was only when the amount of data removed was larger than 80% of the original data that the performance decrease became much steeper. This effect was visible in both DNN and Macau and it was not dependant on the hyperparameters of the model or the specific data that were seen by the models. It was also observed in both classification and regression problems. As the mathematical underpinnings of the two methods are so different, our results suggest that it is the multitask character that drives the benefits of these techniques for dealing with sparse data.

The comparison of the data removal models on the PKIS data set seems to lend further support to this hypothesis, as the performance progression is quite different between the models that generate complete but smaller data set and the label removal model that generates sparse data sets. Performance is higher in the label removal model, where the removed activity labels might be compensated by activity values of the same compound on related assays. This is not possible in the other removal models, as whole compounds or assays are removed. However, the performance progression on the HTSFP subsets for label and compound removal models is very similar.

One explanation for the difference between the data removal models on the two data sets could be the small number of compounds in the PKIS data set, as the effect of the removal of compounds would have a bigger impact on this data set. It does not seem to be connected to differences in the chemical diversity of the compounds in each data set. In both data sets, the average and median similarity between all pairs of compounds are very similar. These results do not allow us to obtain a clear answer on this aspect and more analysis would be needed to fully ascertain what is behind the difference in performance progression between the data sets.

Our results are consistent with other recent analyses of multitask learning on DNNs which have shown that the benefit of multitask DNNs seems to arise when there is mathematical correlation between the test set of one task and the training set of another [18]. It is likely that when individual activity annotations are removed, correlated values from similar assays remain in the data set and are the reason that the loss in performance is not linear. This could be another explanation for the difference between the data removal models on the two data sets. There could be differences in the correlation between the assays when the data is removed.

One of the more surprising results of this analysis was the comparison of DNNs and Macau. The results on the PKIS data set show very similar performance on the full training set, as well as very similar performance progression. However, results on the HTSFP subsets were less favourable for Macau. This may be because we are using a regression technique to simulate classification rather than a true classification technique. It is likely that a thorough exploration of hyperparameters would change the difference in performance on the PKIS set between the two techniques. However, the objective of this work was not to achieve the highest possible performance for any model, and therefore an exhaustive search and optimization of these two methods was not carried out.

One advantage of Macau is that it does not require a GPU to train a model in a reasonable time frame and the implementation used in this work was able to parallelize the computation across different CPU cores to speed up the process. Although GPUs have become more widely available in workstations and high performance computing clusters, they are still less prevalent than CPUs. Therefore, we would encourage research groups to try Macau for multitask learning before investing in a GPU. In our PKIS results, which represented the fairest comparison between the two methods as it was a regression problem, the difference between Macau and DNNs was surprisingly small. Macau also exhibited robustness to sparse data.

The comparison of these novel methods to more established multitask methods in use in the chemoinformatics field, such as Random Forest, is of great interest. However, implementations we had available were not able to handle missing activity data. Because of that, we were able to perform only a limited comparison to Random Forest, which showed similar performance trends to DNN and Macau.

Our results provide a first approximation of how much data is required to carry out effective multitask modelling. However, it is unlikely that missing activity labels in real sparse data sets follow a random distribution. Therefore, it is not possible to assure that the results seen here reproduce what would be seen in real data sets. It would be interesting to see if our methodology could be applied to large and complete activity matrices that have grown over time. In this setting, a better approximation of how much data is required could be obtained. However, we did not access to this type of data to use in the study.

Our analysis shows that it is not necessary to have a complete data set to obtain good results. Indeed, the difference in the performance we obtained between training on the complete data and data with half of its activity labels removed was very small. It brings an interesting counter argument to the common perception that “more data is better”. While it is true that performance on the complete training set was better, it would be interesting to look at how cost effective the improvement is compared to the cost of additional experimental testing.

## Conclusion

Multitask modelling is becoming increasingly prevalent in chemoinformatics, following the popularity of deep neural networks. Data sets extracted from public sources are frequently sparse, but little research has been done to test how performance is affected by the missing data. To explore this issue, we have used two complete data sets to simulate sparseness by removing activity labels progressively. We tested two methodologically distinct multitask techniques on these data sets. Our results show that the performance decrease is at first slow as training data is removed. The rate of performance decrease accelerates after 80% of the training data is deleted. This behaviour is seen in all data sets and techniques we tested. Our work also shows that Macau, a novel technique in the chemoinformatics field, provided very similar results to DNN in our regression tests, and would be of interest to groups performing multitask modelling without access to large GPU computing resources. We were also able to partially compare these novel techniques to a more established one, Random Forest, and the performance progression was similar between all three techniques. Our analysis provides a first estimate of the amount of performance lost due to missing data during training, that is, how much data is required for an effective multitask learning.

## Additional file

**Additional file 1.** Equations of the performance measures used. **Figure S1.** Models for training data removal. **Figure S2.** Effect of varying the random seed values on the PKIS data set. **Figure S3.** Effect of varying the random seed values on HTSFP subsets. **Tables S1–6.** Hyperparameter search values for each technique and dataset. **Tables S7–12.** Hyperparameters sets used for each technique and dataset.
