# Supplementary material for: Effect of missing data on multitask prediction methods
Source: J Cheminform. 2018 May 22;10:26. doi: 10.1186/s13321-018-0281-z (PMC5964064; doi:10.1186/s13321-018-0281-z)
Supplement: Supplementary file 1 — Additional file 1. Equations of the performance measures used. Figure S1. Models for training data removal. Figure S2. Effect of varying the random seed values on the PKIS data set. Figure S3. Effect of varying the random seed values on HTSFP subsets. Tables S1–6. Hyperparameter search values for each technique and dataset. Tables S7–12. Hyperparameters sets used for each technique and dataset. [file 13321_2018_281_MOESM1_ESM.pdf]

Supplemental information

Equations of the performance measures used

For regression models, y_i corresponds to the real value for molecule i and f_i to the predicted value. $\bar{y}$ corresponds to the mean value of the set. The square of the correlation coefficient (ρ^2), the coefficient of determination (R^2), the mean absolute error (MAE), and the root mean square deviation ($RMSD$) were calculated with the following formulae:

$$\rho^2 = \left(\frac{cov(y,f)}{\sigma_y \sigma_f}\right)^2 = \left(\frac{\sum(f_i - \bar{f})(y_i - \bar{y})}{\sqrt{\sum(y_i - \bar{y})^2}\sqrt{\sum(f_i - \bar{f})^2}}\right)^2$$

$$R^2 = 1 - \frac{\sum(y_i - f_i)^2}{\sum(y_i - \bar{y})^2}$$

$$MAE = \frac{1}{n}\sum|y_i - f_i|$$

$$RMSD = \frac{1}{n}\sqrt{\sum(y_i - f_i)^2}$$

For classification, first the numbers of true positives (TP), false positives (FP), false negatives (FN) and true negatives (TN) were calculated by comparing the predicted labels to the real ones. Based on these four values, the precision, the recall, the F_1 score, and the Matthews correlation coefficient (MCC) were obtained:

$$precision = \frac{TP}{(TP + FP)}$$

$$recall = \frac{TP}{(TP + FN)}$$

$$F_1\ score = \frac{2 \cdot (precision \cdot recall)}{(precision + recall)}$$

$$MCC = \frac{TP \cdot TN - FP \cdot FN}{\sqrt{(TP + FP)(TP + FN)(TN + FP)(TN + FN)}}$$

Supplemental Figures

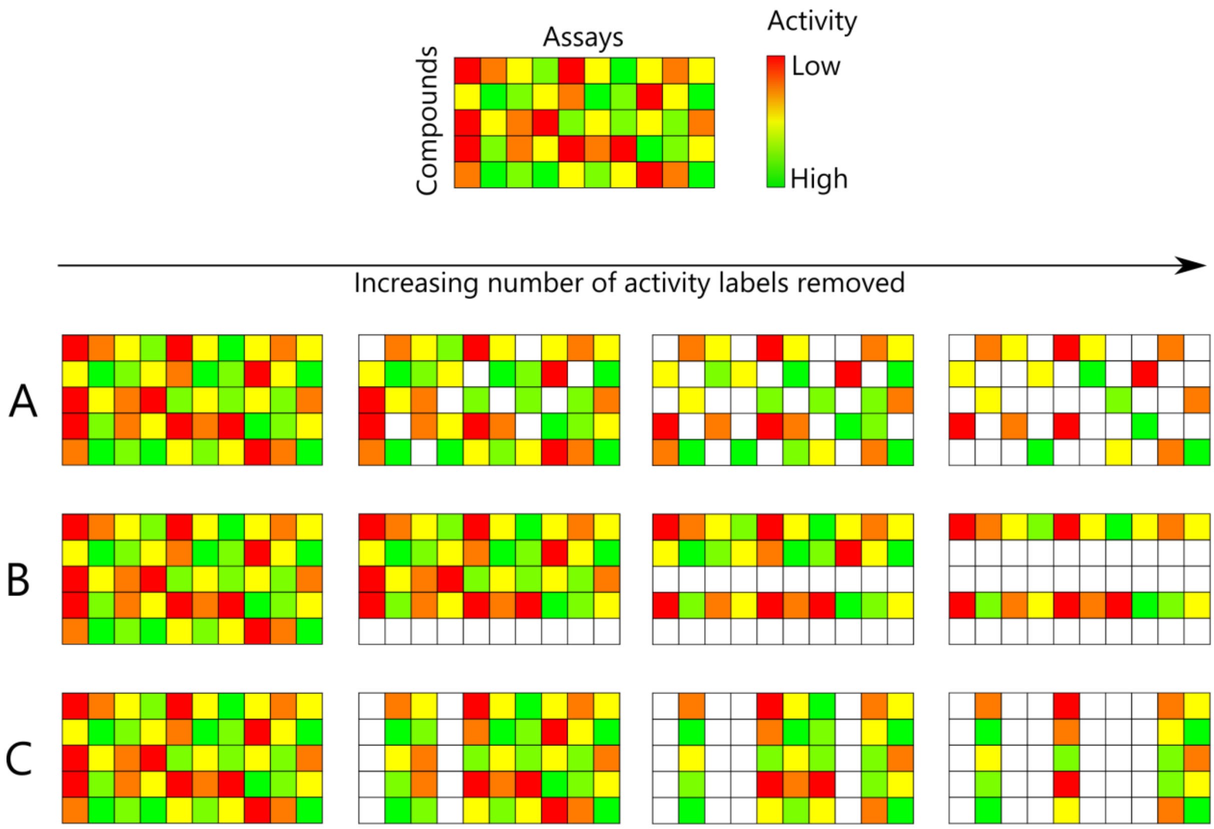

Figure S1. Models for training data removal. The training data set is modelled as a matrix where the compounds are the rows and each assay is a column. Activity labels are removed based on two different models: (A) label removal, where individual cells are removed; (B) compound removal, where whole compounds are removed; and (C) assay removal, where whole assays are removed.

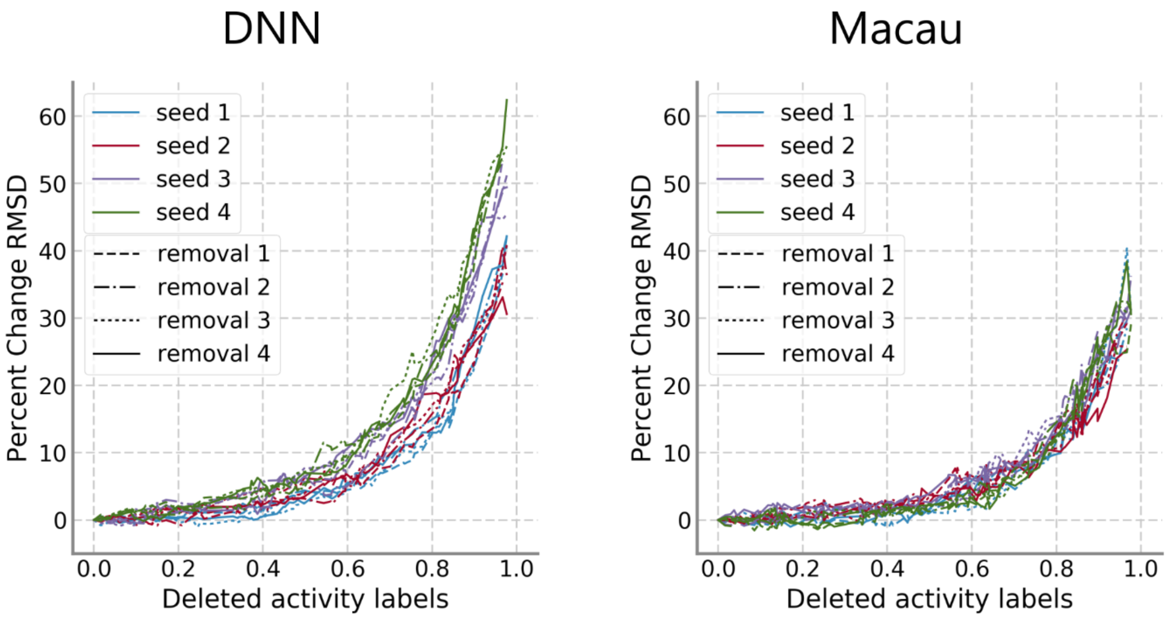

Figure S2. Effect of varying the random seed values on the PKIS data set. Median RMSD values relative to the model with complete training data are shown. The seed applied to the training/test split is shown with colors and the seed of the activity label removal using different line styles. Results for both DNN (left) and Macau (right) are shown.

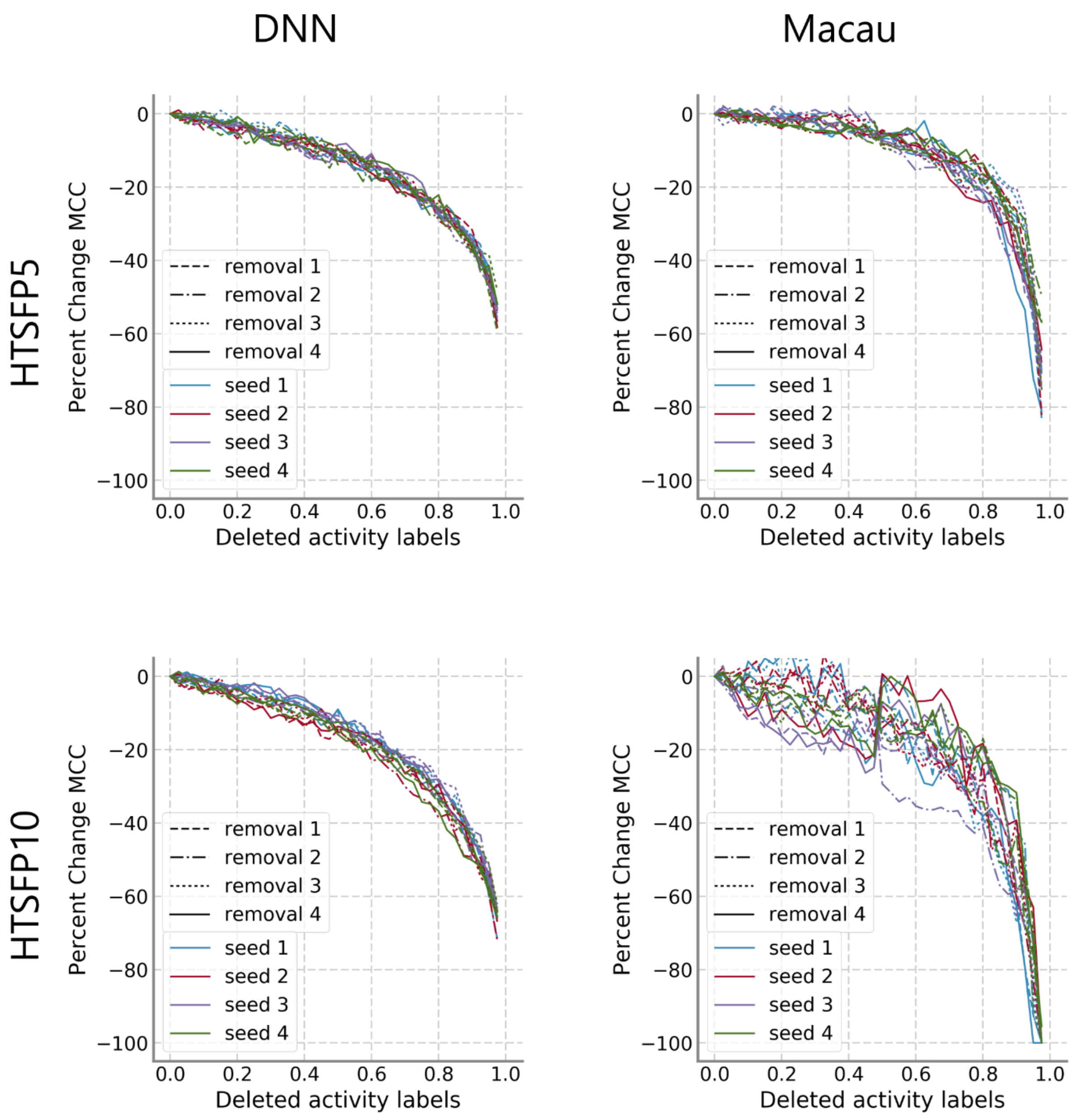

Figure S3. Effect of varying the random seed values on HTSFP subsets. Median MCC values relative to the model with complete training data are shown. The representation of seed values follows the description in Figure S2. Results for both DNN (left) and Macau (right), as well as results for HTSFP5 (top) and HTSFP10 (bottom), are shown.

Supplemental Tables

Table S1. Hyperparameter search values for DNN on the PKIS data set

Hyperparameters	Values
Number of layers	3-5
Number of neurons per layer	5000-10000
Amount of dropout	0.1-0.3
Number of training steps	20000-30000
Size of mini-batch	10-50
Activation function	relu, sigmoid

Table S2. Hyperparameter search values for DNN on the HTSFP data set

Hyperparameters	Values
Number of layers	5-9
Number of neurons per layer	5000-10000
Amount of dropout	0.1-0.3
Number of training steps	150000-250000
Size of mini-batch	64, 128, 256

Table S3. Hyperparameter search values for Macau on the PKIS data set

Hyperparameters	Values
Number of latent dimensions	15-35
Amount of burnin	100-1000
Number of samples	1000-3000
Use of univariate sampler	yes, no

Table S4. Hyperparameter search values for Macau on the HTSFP data set

Hyperparameters	Values
Number of latent dimensions	15-100
Amount of burnin	100-1000
Number of samples	1000-3000
Use of univariate sampler	yes, no

Table S5. Hyperparameter search values for Random Forest on the PKIS data set

Hyperparameters	Values
Maximum number of features	sqrt, log2
Number of trees	1-1000
Bootstrap during tree generation	yes, no

Table S6. Hyperparameter search values for Random Forest on the HTSFP data set

Hyperparameters	Values
Maximum number of features	sqrt, log2
Number of trees	1-1000
Bootstrap during tree generation	yes, no

Table S7. DNN hyperparameters sets used for the PKIS data set

Set #	Minibatch Size	Dropout rate	Number of layers	Number of neurons per layer	Number of training steps
1	41	0.134	4	7050	25705
2	11	0.277	3	6862	26509
3	26	0.113	5	7255	21678
4	49	0.267	4	7160	21209
5	49	0.201	3	8113	21995
6	39	0.181	3	8901	28906
7	36	0.247	3	8306	22730
8	49	0.213	3	6562	25162
9	49	0.150	5	6250	20782
10	24	0.159	3	8677	21136

Table S8. DNN hyperparameters sets used for the HTSFP data set

Set #	Minibatch Size	Dropout rate	Number of layers	Number of neurons per layer	Number of training steps
1	256	0.157461	5	5321	166172
2	64	0.197598	6	6493	182037
3	64	0.11317	6	8086	215474
4	64	0.102579	9	6157	221979
5	64	0.185809	5	5889	157521
6	128	0.216177	8	6692	189536
7	128	0.188179	7	6220	232597
8	128	0.249798	6	5207	195849
9	64	0.293186	5	6071	221968
10	64	0.245538	5	6125	198525

Table S9. Macau hyperparameters sets used for the PKIS data set

Set #	Burnin iterations	Number of samples	Latent space size	Univariate
1	473	2021	25	FALSE
2	625	1092	30	FALSE
3	235	2013	32	TRUE
4	205	1855	33	TRUE
5	665	2409	25	FALSE
6	143	1571	30	TRUE
7	365	2715	29	TRUE
8	343	1173	23	FALSE
9	953	2941	20	FALSE
10	896	2012	33	FALSE

Table S10. Macau hyperparameters sets used for the HTSFP data set

Set #	Burnin iterations	Number of samples	Latent space size	Univariate
1	315	1793	96	FALSE
2	851	2136	16	FALSE
3	365	1141	38	FALSE
4	848	1453	69	FALSE
5	316	1015	13	FALSE
6	416	2455	69	FALSE
7	231	1143	80	FALSE
8	895	1704	67	FALSE
9	774	1471	65	FALSE
10	695	1618	13	FALSE

Table S11. Random Forest hyperparameters sets used for the PKIS data set

Set #	Max features	Number of trees	Bootstrap
1	sqrt	117	FALSE
2	log2	255	FALSE
3	log2	150	FALSE
4	log2	161	FALSE
5	sqrt	310	TRUE
6	log2	663	FALSE
7	log2	889	FALSE
8	sqrt	709	FALSE
9	sqrt	218	TRUE
10	log2	923	FALSE

Table S12. Random Forest hyperparameters sets used for the HTSFP data set

Set #	Max features	Number of trees	Bootstrap
1	sqrt	896	FALSE
2	log2	964	FALSE
3	log2	867	TRUE
4	log2	421	TRUE
5	sqrt	151	FALSE
6	log2	470	FALSE
7	sqrt	740	FALSE
8	sqrt	493	FALSE
9	sqrt	731	FALSE
10	log2	713	TRUE
